# Impact of COVID-19 pandemic on mobility in ten countries and associated perceived risk for all transport modes

**DOI:** 10.1371/journal.pone.0245886

**Published:** 2021-02-01

**Authors:** Diego Maria Barbieri, Baowen Lou, Marco Passavanti, Cang Hui, Inge Hoff, Daniela Antunes Lessa, Gaurav Sikka, Kevin Chang, Akshay Gupta, Kevin Fang, Arunabha Banerjee, Brij Maharaj, Louisa Lam, Navid Ghasemi, Bhaven Naik, Fusong Wang, Ali Foroutan Mirhosseini, Sahra Naseri, Zhuangzhuang Liu, Yaning Qiao, Andrew Tucker, Kasun Wijayaratna, Prince Peprah, Solomon Adomako, Lei Yu, Shubham Goswami, Hao Chen, Benan Shu, Amir Hessami, Montasir Abbas, Nithin Agarwal, Taha Hossein Rashidi

**Affiliations:** 1 Department of Civil and Environmental Engineering, Norwegian University of Science and Technology, Trondheim, Trøndelag, Norway; 2 School of Highway, Chang’an University, Xi’an, Shaanxi, China; 3 Italian Society of Cognitive Behavioral Therapy (CBT-Italy), Firenze, Toscana, Italy; 4 Centre for Invasion Biology, Department of Mathematical Sciences, Stellenbosch University, Matieland, South Africa; 5 Biodiversity Informatics Unit, African Institute for Mathematical Sciences, Cape Town, South Africa; 6 Department of Civil Engineering, Federal University of Ouro Preto, Ouro Preto, Minas Gerais, Brazil; 7 Department of Geography, Lalit Narayan Mithila University, Darbhanga, Bihar, India; 8 Department of Civil and Environmental Engineering, University of Idaho, Moscow, Idaho, United States of America; 9 Indian Institute of Technology Roorkee Department of Civil Engineering, Transportation Engineering Group, Roorkee, Uttarakhand, India; 10 Department of Geography, Environment, and Planning, Sonoma State University, Rohnert Park, California, United States of America; 11 Department of Civil Engineering, Indian Institute of Technology Guwahati, Guwahati, Assam, India; 12 Department of Geography, University of KwaZulu-Natal, Durban, KwaZulu, South Africa; 13 School of Health, Federation University Australia, Berwick, Victoria, Australia; 14 Department of Civil Chemical Environmental and Materials Engineering, University of Bologna, Bologna, Emilia-Romagna, Italy; 15 Department of Civil Engineering/Russ College of Engineering & Technology, Ohio University, Athens, Ohio, United States of America; 16 State Key Laboratory of Silicate Materials for Architectures, Wuhan University of Technology, Wuhan, Hubei, China; 17 School of Medicine, Bam University of Medical Sciences, Bam, Kerman, Iran; 18 School of Mechanics and Civil Engineering, China University of Mining and Technology, Jiangsu, China; 19 Connecticut Transportation Safety Research Center, University of Connecticut, Storrs, Connecticut, United States of America; 20 School of Civil and Environmental Engineering, University of Technology Sydney, Ultimo, New South Wales, Australia; 21 Department of Social Policy Research Centre, University of New South Wales, Sydney, New South Wales, Australia; 22 Department of Engineering and Science, University of Agder, Grimstad, Agder, Norway; 23 School of Civil Engineering, Sun Yat-sen University, Guangzhou, Guangdong, China; 24 Department of Civil Engineering, Indian Institute of Science Bangalore, Bangalore, Karnataka, India; 25 Foshan Transportation Science and Technology Co. Ltd., Foshan, Guangdong, China; 26 Department of Civil and Architectural Engineering, Texas A&M University–Kingsville, Kingsville, Texas, United States of America; 27 Department of Civil and Environmental Engineering, Virginia Tech, Blacksburg, Virginia, United States of America; 28 Department of Civil & Coastal Engineering, University of Florida, Gainesville, Florida, United States of America; Qazvin University of Medical Sciences, ISLAMIC REPUBLIC OF IRAN

## Abstract

The restrictive measures implemented in response to the COVID-19 pandemic have triggered sudden massive changes to travel behaviors of people all around the world. This study examines the individual mobility patterns for all transport modes (walk, bicycle, motorcycle, car driven alone, car driven in company, bus, subway, tram, train, airplane) before and during the restrictions adopted in ten countries on six continents: Australia, Brazil, China, Ghana, India, Iran, Italy, Norway, South Africa and the United States. This cross-country study also aims at understanding the predictors of protective behaviors related to the transport sector and COVID-19. Findings hinge upon an online survey conducted in May 2020 (N = 9,394). The empirical results quantify tremendous disruptions for both commuting and non-commuting travels, highlighting substantial reductions in the frequency of all types of trips and use of all modes. In terms of potential virus spread, airplanes and buses are perceived to be the riskiest transport modes, while avoidance of public transport is consistently found across the countries. According to the Protection Motivation Theory, the study sheds new light on the fact that two indicators, namely income inequality, expressed as Gini index, and the reported number of deaths due to COVID-19 per 100,000 inhabitants, aggravate respondents’ perceptions. This research indicates that socio-economic inequality and morbidity are not only related to actual health risks, as well documented in the relevant literature, but also to the perceived risks. These findings document the global impact of the COVID-19 crisis as well as provide guidance for transportation practitioners in developing future strategies.

## Introduction

The COVID-19 (coronavirus disease 2019) pandemic presents a major challenge for all of humanity and is a huge calamity of the 21^st^ century [1]. Initially spreading in China [2] and facilitated by our hypermobile society and transportation hubs [3–6], this new highly transmissible respiratory syndrome severely broke out in Italy and Iran in March 2020 and then spread at extraordinary rates to other countries, with the United States having the most number of infected patients by May 2020 [7].

The lack of vaccine or clinically effective medical interventions have prompted unprecedented health, social and economic challenges and disruptions [8, 9], thus stressing the need for reshaping the overall design of global resilience [10]. As the greatest risk for infectious diseases spreading within shared travel modes, such as air travel and mass transit, is related to the fact that individuals are in close proximity in a confined environment [11, 12], a number of mobility restrictions have been enacted in most countries to slow down the transmission of the COVID-19 (i.e., social distancing, complete/partial lockdown, required/voluntary quarantining and closure of schools and workplaces) and ease the pressure on health facilities [13–15].

Notwithstanding the “Stay-At-Home (SAH)” message promoted across the globe and the “Work-From-Home (WFH)” reality subsequently achieved whenever possible [16], it is still unclear to what extent individuals have modified their attitude in response to the bans on free movement [17]. As mobility is closely connected to regular habits and reproducible patterns [18], the restrictive measures can represent a “game changer” for all of society entailing permanent behavioral effects comparable to life events and structural shifts among travel modes [19, 20]. Disease risk perceptions are a critical component for properly understanding behavioral changes and altered travel patterns [21–23].

The overarching goal of this research is to shed light on the social and mobility implications of COVID-19 on passenger travels for all transport modes (walk, bicycle, motorcycle, car driven alone, car driven in company, bus, subway, tram, train, airplane) by investigating (i) the effect of localized travel restrictions for three main travel purposes (work/education, free-time and leisure) and (ii) the socio-economic predictors connected to perceived risks. Accordingly, people’s travel behaviors before and during the restrictions and their perceptions concerning the transport sector and the pandemic risks have been assessed in ten countries on six continents with a web-based survey: Australia, Brazil, China, Ghana, India, Iran, Italy, Norway, South Africa and the United States (hereafter, also referred to by their acronyms AU, BR, CH, GH, IN, IR, IT, NO, ZA and USA, respectively). Moreover, as the consequences of COVID-19 can be compared to the effects engendered by a large-scale natural disaster [24], this study includes both developed and developing countries across six continents in order to characterize the impact in different socio-economic contexts [25–27].

The evaluation of perceived risks in the transport sector due to the COVID-19 pandemic is performed in this study pivoting on the Protection Motivation Theory. This major social psychological model was developed by Ronald Rogers [28, 29] and accounts for how individuals modify attitudes and behavioral styles when interpreting (threat appraisal) and reacting (coping appraisal) to fear appeals and stressful stimuli. Furthermore, the perceived risk, regarded as a distal predictor of intentions and behavioral change, is a cornerstone of the Health Belief Model [30–32], which explains the likelihood of engagement in health-promoting behavior in response to stimuli or cues to action.

Considering the existing literature, few studies have focused on measuring the impacts of COVID-19 on individuals on a multi-country scale simultaneously. The large international data collection efforts have been dedicated to consumer sentiment in thirty-four countries [33], personal beliefs about the pandemic across six countries [34], individual protective measures in twenty-one countries [35] and mental well-being across fifty-eight countries [36]. The findings of this investigation can be used for documenting the impacts of the COVID-19 crisis on a global scale.

## Materials and methods

This study collected all the empirical data by means of an online survey. The information regarding the individual mobility patterns and the travel perceptions were gathered deploying matrix-level and Likert-type scales questions [37]. The structure of the survey [38] can be subdivided into seven main parts: Introductory Part, Part A, Part B, Part C, Part D, Part E and Part F. In the Introductory Part respondents were asked about their demographic profile (age, gender, location, education), whether they worked/studied remotely (Work-From-Home, WFH) because of the restrictions and their Commuting Distance (CD). The frequency of use of each transport mode during and before the enactment of the restrictions was investigated in Part A (travels related to work/study) and Part B (travels related to free time). Part C dealt with the frequency of four activities, namely purchasing essential goods, purchasing nonessential goods, visiting relatives and joining social gatherings. The answers respondents could choose from in Part A, Part B and Part C were “more than 3 times per week”, “2–3 times per week”, “1 time per week”, “2–3 times per month”, “1 time per month”, “less than 1 time per month”, “never”. The survey also focused on understanding the risk perceptions encompassing the transport sector and the pandemic according to the Protection Motivation Theory as three Likert-type queries (Part D, Part E, Part F) pivoted on three corresponding dimensions: perceived probability of contracting COVID-19 for different transport modes (vulnerability appraisal, Part D), perceived effectiveness of the associated restrictions to limit the virus transmission (response efficacy, Part E) and expected time for the transportation sector to recover (level of confidence, Part F). The perceived probability of contracting COVID-19 for different transport modes (Part D) and the perceived effectiveness of the associated restrictions to curb the virus transmission (Part E) were assessed using two 7-point Likert-type scales ranging from 1 to 7, where the lower and upper limits represent “extremely low” and “extremely high”, respectively (responses in Part D) or “extremely ineffective” and “extremely effective”, respectively (responses in Part E). Furthermore, the participants provided their anticipated recovery time of the transportation system in their region/province/state, in their country and in the world (Part F) according to the following options: less than 6 months, between 6 and 12 months, between 12 and 18 months, between 18 and 24 months and more than 24 months; each response has been associated with a discrete value ranging from 1 to 5. The overall structure of the survey is reported in Table 1.

**Table 1:** Survey structure.

PART	TOPIC
Introductory Part	Demographics, Work-From-Home (WFH), Commuting Distance (CD)
Part A	Frequency of use for each transport mode related to work/study travels
Part B	Frequency of use for each transport mode related to free time travels
Part C	Frequency of four mobility purposes
Part D	Perceived probability of contracting the virus for each transport mode
Part E	Perceived effectiveness of the restrictions for each transport mode
Part F	Expected time for the transportation sector to recover

Parts composing the survey and topics dealt with [38].

The survey was distributed between the 11^th^ and the 31^st^ of May 2020 in Australia, Brazil, China, Ghana, India, Iran, Italy, Norway, South Africa and the United States. The questionnaire was created with Google Forms and WenJuanXing and the same content was conveniently translated into Chinese, English, Italian, Norwegian, Persian and Portuguese. Overall, all the survey questions were designed to be brief and concise. The linguistic validity across the ten countries was pursued following a translation-back-translation approach [39]: after translating the survey into local languages, the survey was back translated. The research team carefully addressed and resolved all the discrepancies to ensure full linguistic equivalence.

The survey was distributed using a combination of purposive and snowball techniques [40, 41]: this included direct emails to personal contacts, university students and posts to social and professional networks including but not limited to Facebook, LinkedIn, Twitter, Instagram, Skype, WhatsApp, WeChat, Weibo, QQ and Douban. Additional respondents were gained via snowball sampling through the forwarding of email invitations by initial recipients and shares of original social media posts (e.g., retweets). Only adult respondents could join the survey and informed written consent was obtained from all of them consistent with the Declaration of Helsinki. The survey study was reviewed and approved by two major institutional review boards, namely Norwegian Centre for Research Data (reference code 332877) and Ohio University Office of Research Compliance (reference code 20-E-199). Overall, 9,394 responses were collected and the formed dataset is publicly available [38].

Notably, COVID-19 has struck different locations with varying intensity at different times. However, based on the COVID-19 response stringency index [42], all the investigated countries had implemented their most restrictive policies by the 11^th^ of May. Therefore, all the survey participants were able to compare their mobility behavior “before” (retrospective questions) and “during” (current questions) the pandemic. Notwithstanding the possible distorted recalls from partially forgotten or telescoped occurrences [43–46], retrospective questions are deemed to produce reliable responses if the investigated time is smaller than one year [47, 48]. Considering that the survey data collection was accomplished in May 2020 and that the curfew measures were enacted in all the investigated countries between January and March 2020 [42], the retrospective questions of this study refer to a temporal span varying from some weeks to very few months; therefore, the questionnaire responses describing the travel behaviors before the enforcement of the restrictions are not likely to be largely biased.

Following the survey, the study conducts statistical analyses to explore the possible correlations between health risks and individual perceptions considering ten key indicators pertaining to three macro socio-economic areas relevant for this international study [49, 50]. Individual variables are gender, education and age. Country-related variables include population density and socio-economic condition as measured by the Gini index [51], Inclusive Development Index [52] and Human Development Index [53]. Additional variables reflect the epidemiological situation in each country as indicated by the reported number of confirmed cases and the reported number of deaths per 100,000 inhabitants [7] and the share of the population working from home, as measured by the survey. The central hypothesis of this study is that income inequality and pandemic-related death toll are the main drivers for changes in cognitive behaviors towards travel and perceived risks.

A Negative Binomial Model (NBM) was the regression method adopted to find the correlations between the response variables for Part D, Part E, Part F and the explanatory variables. NBM regression is a Generalized Linear Model and it was chosen as the hypotheses necessary to perform simpler analyses (i.e., ANOVA or linear regression) were not fulfilled (such as normality of the residuals) [54, 55]. Gender and education were treated as the categorical explanatory variables, while all the other factors as continuous ones. All the statistical analyses were accomplished with the software package IBM SPSS Statistics version 26.

## Results and discussion

### Survey outreach

**Fig 1:**
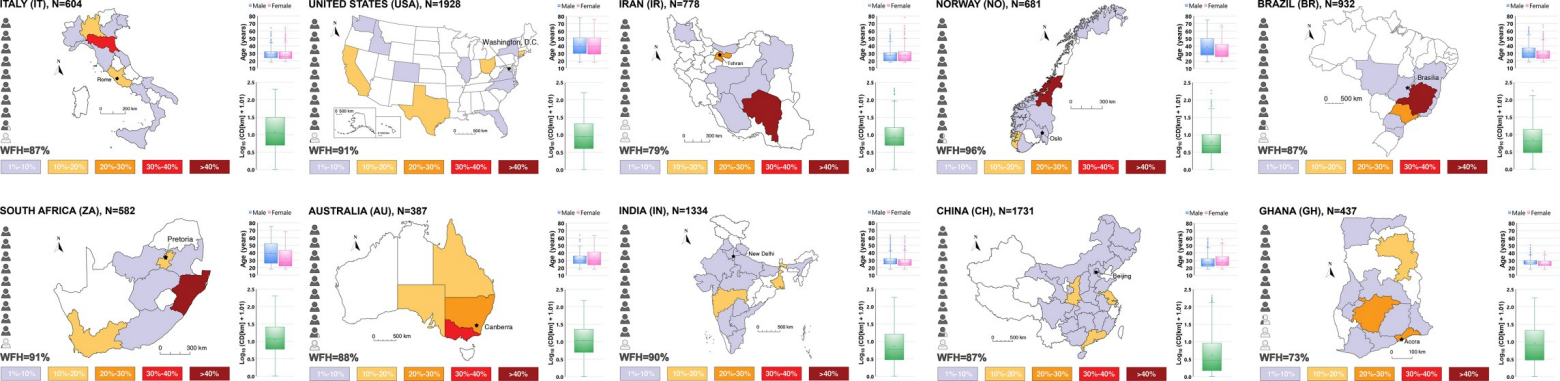
Survey outreach. Sample size, geographical distribution of respondents within each country (percent), gender split, median age, Commuting Distance (CD) under pre-pandemic conditions and Work-From-Home (WFH) rate. Maps are adapted for illustrative purpose from images that are publicly available according to Creative Commons 4.0 License [59–68].

The geographical distribution and the demographic information of the sample are reported in Fig 1. Overall, the survey included a balanced representation by gender (male 50.9% and female 48.9%) with total of 9,394 participants. Respondents tended to be younger and middle-aged adults (32.6 ± 11.6, for mean ± SD hereafter) and were also notably comprised by those with higher levels of education (81.3% held at least a bachelor's degree). Thus, the results here likely reflected changes in behavior and perceptions among upper classes, particularly in the less wealthy countries where internet access to the online-administered survey was less ubiquitous. In all of the Figures and Tables, the countries are listed according to decreasing reported number of pandemic-related deaths per 100,000 inhabitants calculated between the 11^th^ and the 31^st^ of May 2020 [7]. During this timeframe corresponding to the survey distribution, the countries with the highest and the lowest reported death tolls per 100,000 individuals were Italy (54.3) and Ghana (0.1), respectively.

At the same time, it must be acknowledged that this study was not exempt from a notable limitation that web-based surveys commonly have [56], especially when considering the need for getting the questionnaire rapidly distributed given the quickly growing disruptions. The survey sample, albeit substantial, cannot be regarded as demographically representative of the entire population in each of the ten countries by age or gender but rather as indicative of the perceptions of the general public; moreover, inadequate internet access may have been encountered in developing countries during the survey. Another relevant circumstance one should be aware of when interpreting the results is the seasonality of mobility [57, 58], as in the northern and southern hemisphere the pre-pandemic season was winter and summer, respectively.

### Changes to travel behavior

The mobility restrictions exerted a major impact on the way in which work is done and its availability. The work or school Commuting Distance (CD) prior to the pandemic (14.6 ± 25.0 km) was essentially evaporated for the largest part of the respondents as 87.1 percentage of the population sampled shifted to an online or remote environment (Work-From-Home, WFH), this is in good accordance with other studies quantifying the refraining from travelling [69, 70]. The lowest WFH rate, although still a supermajority, was found in Ghana (73.2%), Norway registered the highest WFH rate (95.7%).

**Fig 2:**
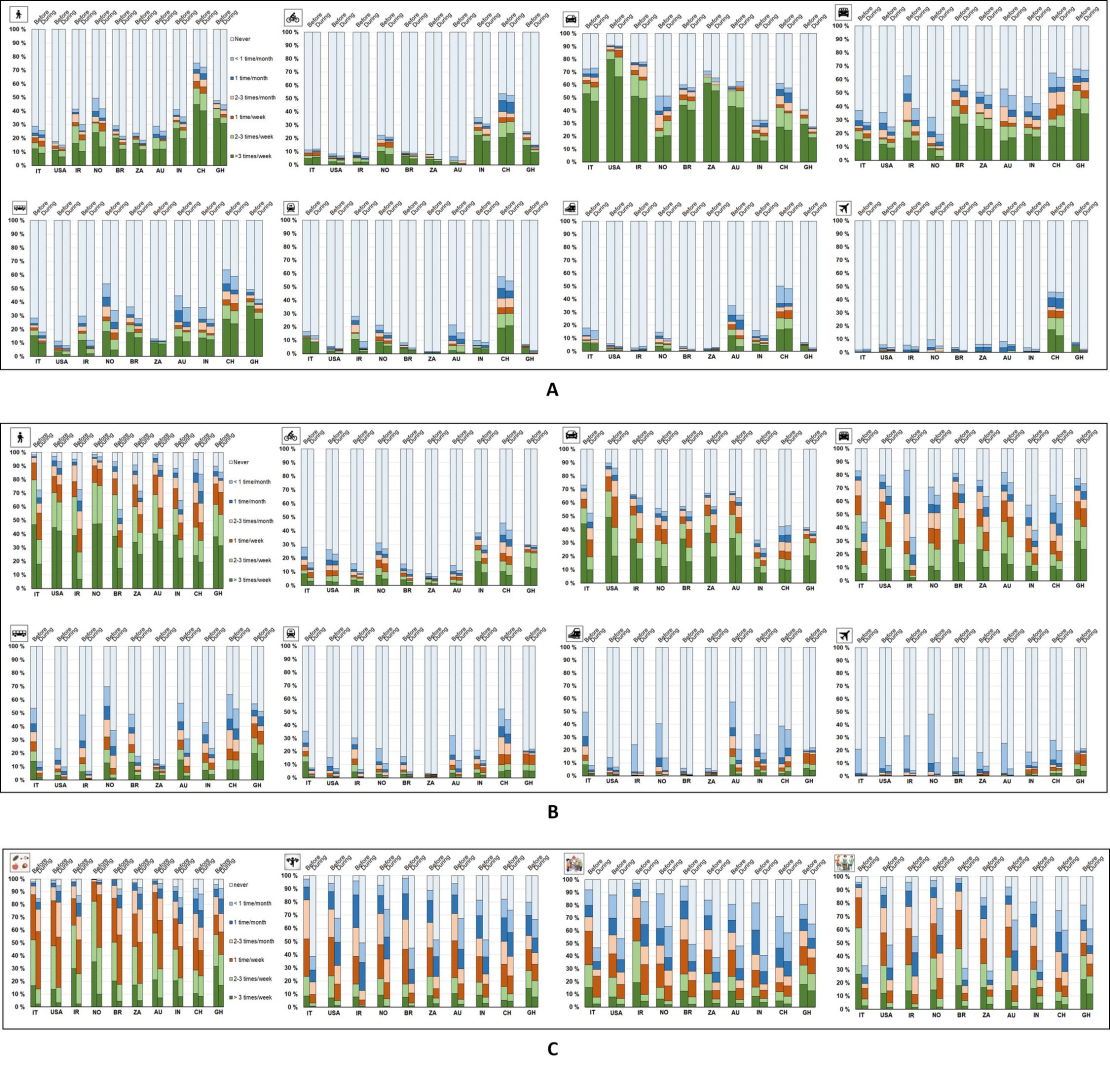
Mobility before and during the implementation of pandemic-related restrictive measures. Mobility related to commuting travels of survey respondents who did not work from home during restrictions (N = 1,210) (A) and mobility related to non-communing travels of all survey respondents (N = 9,394) during free time (B) and for different purposes (C).

The restrictive measures have massively impacted individual mobility patterns related to both commuting and non-commuting travel for all transport modes considered in the study as depicted in Fig 2A and 2B, respectively. The results displayed in Fig 2A only take into consideration the respondents who, notwithstanding the restrictions, did not work from home during the COVID-19 pandemic (12.9% of the population sampled). The use frequency of non-public transport modes has different degrees of change across the surveyed countries; the largest increases in the number of “never” responses were registered in Iran for walk (+11.3%) and in Ghana for cycle (+10.2%) and car driven alone (+13.7%). Depending on the country, substantial changes were also found for public transportation; the most significant hikes in the quantity of “never” responses were obtained in Iran for subway/tram (+18.7%), in Australia for train (+7.2%) and in Norway for car driven in company (+12.6%), bus (+19.4%) and airplane (+4.9%). Considering average values assessed across the countries, the transport modes facing the largest and smallest increase in the amount of “never” responses were bus and car driven in company (+9.0% and +7.7%) on the one hand and airplane and car driven alone (+1.9% and +2.1%) on the other. The most significant reductions in the quantity of “more than 3 times per week” were related to walk (-5.4%) and bus (-5.4%).

Furthermore, regardless of the pandemic, the findings are useful to characterize the commuting patterns in the surveyed countries. It is evident that the habits of respondents from China, India and Ghana cover the entire modal range as already observed elsewhere [71], while people in the United States prefer driving car at the expense of public transportation. Furthermore, a significant use of airplanes was found for respondents in China; which is a determining feature for the trend of the global air travel market [72].

Fig 2B refers to the non-commuting mobility of the entire population sampled. Across all countries, use of all transport modes generally plummeted. Considering average values estimated for all the respondents, the amount of people reporting they never used bus, car driven in company, airplane, train had a remarkable hike of +22.7%, +16.2%, +16.0%, +15.6% respectively. The share of respondents saying they never walked during the pandemic increased by 15.5%, implying that a substantial proportion of people drastically reduced simply going outside. Reductions in use of any mode varied by country. Italy (+27.3%) and Iran (+22.1%), two countries hit hard relatively early in the pandemic, saw the greatest increases in respondents stating they did not use any of the transport modes. Conversely, the smallest decreases in travel were registered in those countries with the lowest pandemic-related death toll per 100,000 inhabitants, namely China (+5.7%) and Ghana (+3.1%). These findings are in line with previous studies shedding light on the negative relationship between pandemics (SARS, Ebola, MERS) and travel demand for both work and recreational purposes [73–75].

Looking at changes to non-commute trips, Fig 2C shows the frequency of four mobility purposes: purchasing essential goods, purchasing nonessential goods, visiting relatives and joining social gatherings. As observed elsewhere [76, 77], even if the restrictions have not significantly modified the amount of individuals reporting “never” response related to buying essential goods (+6.0%), people did so less frequently than before the pandemic: the quantity of “more than 3 times/week” and “2–3 times/week” responses decreased by -14.9% and -12.8%, respectively. Considering each country, the biggest reductions referring to the amount of “more than 3 times/week” responses were observed in Iran (-27.6%) and Norway (-25.3%). In line with the findings reported in Fig 2B, people drastically reduced the mobility associated with free-time travels: notably, approximately 50% of the respondents in Italy, Brazil, South Africa and India never went out to purchase nonessential goods, visit relatives or hang out with friends. Referring to these three mobility activities assessed across the studied countries, the average increase in the number of “never” responses was +32.0%, +31.4%, +39.3%, respectively. Similar to the results discussed in Fig 2B, the least significant variations were registered in the countries characterized by the smallest death toll per 100,000 inhabitants, namely China (+12.0%) and Ghana (+15.6%). Overall, in line with aggregate findings evaluated by Information Technology companies [78, 79] and application software [80], the COVID-19 restrictive measures were highly effective to limit and radically alter mobility via every mode and for any purpose.

### Transportation perceptions

**Fig 3:**
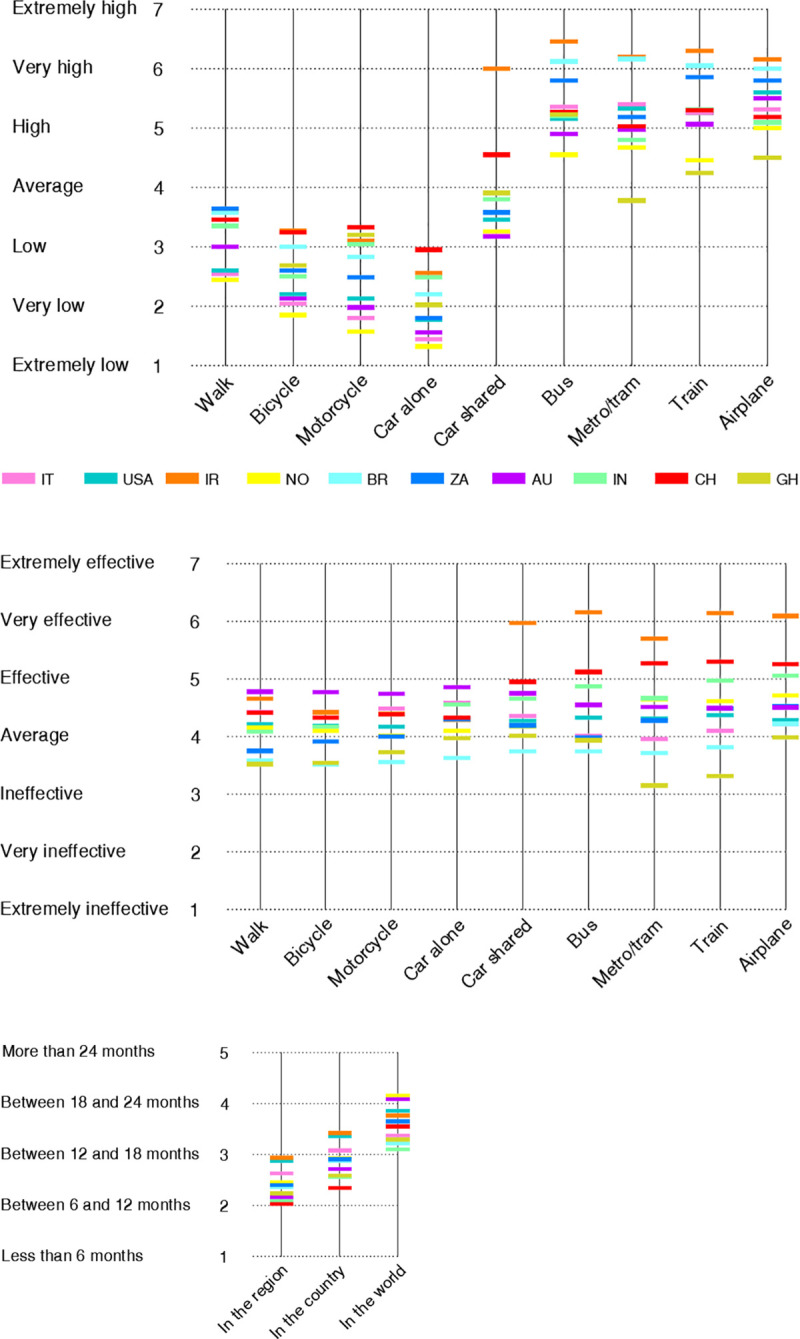
Perceptions encompassing mobility and pandemic. Perceived probability of contracting COVID-19 (A) and perceived effectiveness of curbing COVID-19 (B) for transport modes according to a Likert-type scale varying from “1 = extremely low/ineffective” to “7 = extremely high/effective”. Perceived time needed by the transportation sector to completely recover (C) according to the scale “1 = less than 6 months”, “2 = between 6 and 12 months”, “3 = between 12 and 18 months”, “4 = between 18 and 24 months”, “5 = more than 24 months”.

The study characterized the risk perceptions related to the transport sector and the pandemic by deploying the three Likert-type queries (Part D, Part E, Part F) formulated according the Protection Motivation Theory. Fig 3 displays the mean value of the responses assessed for each country. The same values with the associated standard deviations are reported in more detail in S1 Table. The internal consistency of the responses collected for Part D, Part E and Part F was high as the values of Cronbach’s alpha were equal to 0.834, 0.888 and 0.820, respectively.

Considering the responses to Part D reported in Fig 3A, the perceived probability of contracting the virus increased when an individual shifted from non-public to public transport modes. The two safest transport modes were deemed to be driving the car alone (2.15 ± 1.44 in the Likert scale) and riding a motorcycle (2.64 ± 1.52). On the other hand, respondents believed that the two most dangerous travel modes were airplane (5.44 ± 1.61) and bus (5.39 ± 1.65). Subway/tram and trains were also seen as relatively risky with mean values on the upper half of the scale. The avoidance of public transport modes is in accordance with previous findings portraying the precautionary behaviors in response to a perceived pandemic threat [81]. In alignment with the Protection Motivation Theory, respondents significantly reduced travel by the modes appraised as the riskiest ones as discussed for Fig 2B.

For Part E on efficacy of the travel restrictions, the average response scores were comprised between 4.16 ± 1.67 for bicycles and 4.83 ± 1.81 for airplanes, respectively. Looking at Fig 3B, the variation in sentiment is quite small, as this may have been one of the harder questions for respondents to answer. Notably, across all modes, it is possible to observe that survey participants from Australia, Iran, China on one hand and from Brazil, Ghana on the other hand were respectively associated with the highest and the lowest response scores. Furthermore, when addressing Part F as displayed in Fig 3C, the respondents believed that the transportation system would recover approximately between 12 and 18 months in their region/province/state (2.43 ± 1.37) and country (2.87 ± 1.39) and between 18 and 24 months worldwide (3.61 ± 1.45).

The Negative Binomial Model (NBM) was employed to find statistically significant correlations between the respondents’ opinions treated as response variables and any of the ten explanatory variables comprising demographic information, socio-economic status and epidemiological situation. Due to collinearity issues, only seven predictors were considered in the analyses: gender, education, age, Gini index, population density, WFH percentage and number of pandemic-related deaths per 100,000 inhabitants for each country. The parameters of the regression and the statistical significance are displayed in Table 2. The likelihood ratio Chi-square and the Deviance/df ratio are reported in S2 Table and in S3 Table, respectively.

**Table 2:** Results from negative binomial model (NBM) regression method.

***Part D*** ^***a***^^,^^***b***^	**Walk**	**Bicycle**	**Motorcycle**	**Car alone**	**Car shared**
**Male | Female**	-.068 ± .025**	-.050 ± .025*	-.048 ± .025^ns^	.020 ± .026^ns^	.012 ± .024^ns^
**Education 1 | 6**	.269 ± .300^ns^	.459 ± .300^ns^	.384 ± .302^ns^	.399 ± .312^ns^	.114 ± .298^ns^
**Education 2 | 6**	.203 ± .120^ns^	.346 ± .120**	.291 ± .121*	.556 ± .121***	.050 ± .118^ns^
**Education 3 | 6**	.129 ± .052*	.088 ± .053^ns^	.065 ± .053^ns^	.130 ± .055*	.048 ± .050^ns^
**Education 4 | 6**	.058 ± .043^ns^	.043 ± .044^ns^	.064 ± .044^ns^	.122 ± .046**	.015 ± .042^ns^
**Education 5 | 6**	.020 ± .044^ns^	.043 ± .045^ns^	.042 ± .046^ns^	.074 ± .047^ns^	.049 ± .043^ns^
**Age**	-.005 ± .001***	-.005 ± .001***	-.005 ± .001***	-.006 ± .001***	-.004 ± .001**
**Gini**	.008 ± .002***	.010 ± .002***	.013 ± .002***	.013 ± .002***	.004 ± .002**
**Deaths**	-.005 ± .001***	-.004 ± .001***	-.005 ± .001***	-.005 ± .001***	-.003 ± .001**
**Density**	.000 ± .0001**	.000 ± .0001***	.001 ± .0001***	.001 ± .0001***	.000 ± .0001^ns^
**WFH**	-.008 ± .003**	-.011 ± .003***	-.019 ± .001***	-.009 ± .003**	-.019 ± .003***
	**Bus**	**Metro/Tram**	**Train**	**Airplane**	
**Male | Female**	-.038 ± .023^ns^	-.040 ± .027^ns^	-.040 ± .025^ns^	-.036 ± .024^ns^	
**Education 1 | 6**	-.211 ± .298^ns^	-.284 ± .340^ns^	-.138 ± .307^ns^	-.180 ± .308^ns^	
**Education 2 | 6**	-.232 ± .117*	-.271 ± .125*	-.152 ± .119^ns^	-.198 ± .119^ns^	
**Education 3 | 6**	-.016 ± .049^ns^	-.068 ± .056^ns^	-.023 ± .051^ns^	-.020 ± .050^ns^	
**Education 4 | 6**	-.033 ± .040^ns^	-.059 ± .046^ns^	-.046 ± .043^ns^	-.036 ± .042^ns^	
**Education 5 | 6**	-.010 ± .041^ns^	-.018 ± .047^ns^	-.008 ± .044^ns^	.002 ± .043^ns^	
**Age**	-.002 ± .001^ns^	-.001 ± .001^ns^	-.001 ± .001^ns^	.000 ± .001^ns^	
**Gini**	.006 ± .002***	.005 ± .002**	.007 ± .002***	.004 ± .002**	
**Deaths**	.000 ± .001^ns^	.002 ± .001*	.001 ± .001^ns^	.001 ± .001^ns^	
**Density**	.000 ± .0001^ns^	.000 ± .0001^ns^	.000 ± .0001^ns^	.000 ± .0001^ns^	
**WFH**	-.008 ± .003**	.001 ± .003^ns^	-.003 ± .003^ns^	.004 ± .003^ns^	
***Part E*** ^***a b***^	**Walk**	**Bicycle**	**Motorcycle**	**Car alone**	**Car shared**
**Male | Female**	-.033 ± .026^ns^	-.046 ± .027^ns^	-.042 ± .027^ns^	-.035 ± .026^ns^	-.001 ± .026^ns^
**Education 1 | 6**	-.093 ± .303^ns^	.125 ± .321^ns^	-.225 ± .346^ns^	.095 ± .309^ns^	-.048 ± .323^ns^
**Education 2 | 6**	.066 ± .120^ns^	.061 ± .121^ns^	.124 ± .120^ns^	.028 ± .123^ns^	.003 ± .120^ns^
**Education 3 | 6**	.037 ± .055^ns^	.023 ± .055^ns^	.014 ± .056^ns^	.017 ± .056^ns^	.033 ± .054^ns^
**Education 4 | 6**	-.017 ± .046^ns^	-.010 ± .047^ns^	-.007 ± .047^ns^	-.004 ± .047^ns^	-.018 ± .045^ns^
**Education 5 | 6**	.003 ± .047^ns^	.009 ± .047^ns^	.001 ± .048^ns^	.012 ± .048^ns^	.013 ± .047^ns^
**Age**	.001 ± .001^ns^	.001 ± .001^ns^	.001 ± .001^ns^	.001 ± .001^ns^	-.001 ± .001^ns^
**Gini**	-.004 ± .002*	-.003 ± .002^ns^	.002 ± .002^ns^	-.002 ± .002^ns^	-.001 ± .002***
**Deaths**	.000 ± .001^ns^	.000 ± .001^ns^	.000 ± .001^ns^	.001 ± .001^ns^	-.001 ± .001^ns^
**Density**	.000 ± .001^ns^	.000± .0001^ns^	.000 ± .0001^ns^	.000 ± .0001*	.000 ± .0001^ns^
**WFH**	-.001 ± .001^ns^	.001 ± .003^ns^	-.001 ± .003^ns^	.001 ± .001^ns^	-.009 ± .003***
	**Bus**	**Metro/Tram**	**Train**	**Airplane**	
**Male | Female**	.019 ± .024^ns^	.004 ± .029^ns^	.009 ± .026^ns^	.007 ± .026^ns^	
**Education 1 | 6**	-.292 ± .317^ns^	-.038 ± .309^ns^	-.254 ± .314^ns^	-.264 ± .325^ns^	
**Education 2 | 6**	-.051 ± .118^ns^	-.121 ± .125^ns^	-.084 ± .120^ns^	-.159 ± .121^ns^	
**Education 3 | 6**	.006 ± .051^ns^	-.061 ± .060^ns^	-.017 ± .055^ns^	-.023 ± .053^ns^	
**Education 4 | 6**	-.043 ± .043^ns^	-.053 ± .050^ns^	-.055 ± .046^ns^	-.069 ± .044^ns^	
**Education 5 | 6**	.014 ± .044^ns^	.009 ± .052^ns^	.008 ± .047^ns^	.002 ± .045^ns^	
**Age**	-.002 ± .001^ns^	-.001 ± .001^ns^	-.002 ± .001^ns^	-.002 ± .001^ns^	
**Gini**	-.003 ± .002^ns^	.000 ± .002^ns^	.000 ± .002^ns^	.001 ± .002*	
**Deaths**	-.002 ± .001**	-.003 ± .001**	-.003 ± .001**	.004 ± .001***	
**Density**	.000 ± .0001^ns^	.000 ± .0001^ns^	.000 ± .0001^ns^	.000 ± .0001^ns^	
**WFH**	-.008 ± .003**	.001 ± .004^ns^	-.005 ± .003^ns^	-.005 ± .003^ns^	
***Part F*** ^***a*,*b***^	**In the region**	**In the country**	**In the world**		
**Male | Female**	-.001 ± .027^ns^	-.052 ± .026*	-.067 ± .025*		
**Education 1 | 6**	.306 ± .324^ns^	.242 ± .318^ns^	.055 ± .313^ns^		
**Education 2 | 6**	.291 ± .127*	.253± .125*	-.015 ± .124^ns^		
**Education 3 | 6**	.050 ± .055^ns^	.079 ± .054^ns^	-.019 ± .052^ns^		
**Education 4 | 6**	-.020 ± .046^ns^	-.038 ± .045^ns^	-.057 ± .043^ns^		
**Education 5 | 6**	.001 ± .047^ns^	-.012 ± .046^ns^	-.031 ± .044^ns^		
**Age**	.005 ± .001***	.004 ± .001**	-.001 ± .001^ns^		
**Gini**	.001 ± .002^ns^	.001 ± .002^ns^	-.004 ± .002*		
**Deaths**	.005 ± .001***	.005 ± .001***	.001 ± .001^ns^		
**Density**	.000 ± .0001**	.000 ± .0001***	.000 ± .0001***		
**WFH**	-.006 ± .003*	-.004 ± .003^ns^	.004 ± .003^ns^		

^a^Superscripts are ns = non-significant

* = p < .05

** = p < .01

*** = p < .001.

^b^Abbreviations are “Education 1” = Primary school, “Education 2” = Middle school, “Education 3” = High school, “Education 4” = BSc, “Education 5” = MSc, “Education 6” = PhD.

Parameters estimates, standard deviation and statistical significance (B ± S.E.^a^) for the responses to perceived probability of contracting COVID-19 (Part D), perceived effectiveness of curbing COVID-19 (Part E) and perceived time needed by the transportation sector to completely recover (Part F).

Considering the significant predictors (p < .05) for the responses to Part D, the perceptions were strongly associated with Gini index and number of deaths per 100,000 individuals. Furthermore, focusing on non-public transport modes, additional predictors came into play: age, population density and WFH percentage. As for the perceived effectiveness associated to travel restrictions (Part E), the COVID-19 death toll per 100,000 individuals was the only significant predictor across nearly all transport modes. Depending on the geographical extension considered, all the predictors were statistically correlated to the results of Part F. Overall, it can be inferred that the perceptions of the entire population strongly hinged upon two factors: Gini index and the reported number of pandemic-related deaths per 100,000 inhabitants.

The correlation between socio-economic status and health is well established in epidemiologic research [82]. Previous compositional studies linking income inequality, measured here by Gini index, to morbidity at high levels of geographical aggregation are documented in population epidemiology [83–85]. The deprivation assessed by income inequality explains how socio-economic gradients influence health and well-being [86, 87], and this has also been accounted for by neo-material interpretation [88]. Income inequality is a determinant of population health, in particular at large scale cross-country analyses [89, 90]. Different from the perceived vulnerability, the actual vulnerability to disease infection has largely been attributed to socio-economic status. Such studies have focused on different types of diseases, mainly HIV, smoking-related complications and pandemics [91–93]. This research sheds new light on the fact that socio-economic inequality, expressed here as Gini Index, and morbidity do not only aggravate actual health risks, as well documented in Protection Motivation Theory, but also perceived risks.

This may indicate accurate appraisal by participants of the risk associated with inequality. In several behavioral models the effect of perceived risk on actual behavior is moderated by one or more factors which capture a person’s ability to control or direct their own behavior [29, 94, 95]. These changes in mobility may reflect not only differences in the perceived risk of various transport modes, but also the availability or ability to choose alternative transport modes [96, 97]. Conversely, higher perceived risk may reflect lower perceived or actual control over exposure to risk [98]. Inequality may therefore affect perceived risk via impacts on the availability of different transport modes.

A consideration must be made regarding the demographic indicators (age, gender, education). The study documents that demographic factors that are often clearly correlated at national level with pandemic-related risk protective and preventive behavior [50, 99, 100] are not statistically significant in this cross-country survey. Observing each predictive variable for all the data related to Part D, Part E and Part F, gender and education are seldom statistically significant.

## Conclusions

As public transport typically brings people into close contact in a confined space, stark mobility restrictions have been dully adopted to flatten the curve of COVID-19 infections, thus forcing entire populations to radically modify their travel behavior and shaping a worldwide change which is unprecedented in human history compared to other recent epidemics (MERS, SARS, Ebola). This research focused on comparing people’s mobility before and during the restrictions and the associated perceived risks in ten countries: Australia, Brazil, China, Ghana, India, Iran, Italy, Norway, South Africa and the United States. These findings can be rendered:

1. The significant mobility disruptions related to the restrictions enforced to tackle the COVID-19 pandemic pertained all transportation modes and all travelling purposes, albeit the extent of the transformations was different for each surveyed country.
2. Using negative binomial regressions, the main socio-economic drivers of change in travel cognitive behaviour and individual perceptions were income inequality, expressed as Gini index, and the reported death toll due to COVID-19 per 100,000 inhabitants.
3. The results of this study indicate that socio-economic inequality and morbidity are not only related to actual health risks, as well documented in Protection Motivation Theory and Health Belief Model literature, but also to the perceived risks.

As the COVID-19 pandemic is likely to entail a long-term effect on transport mode choice and people’s cognitive assessment towards travel, transit operators need to carefully take into consideration the modal split changes and, regardless of socio-economic inequalities, endeavor to gain public trust and make journeys less risky by interpreting the pandemic as a “catalyst for change” [101] and “hallmark of recovery” [102]. The findings of this study can provide guidance for transport practitioners and policy makers to develop mobility strategies and intervention mechanisms to combat the current crises and future pandemics facilitating the interventions according to a user’s perspectives. Further research including replications of such behavioral surveys and the analysis of empirical travel data is necessary to understand longer term implications.

## Supporting information

S1 Table Perceptions encompassing mobility and pandemic. Perceived probability of contracting COVID-19 (Part D) and perceived effectiveness of curbing COVID-19 (Part E) for transport modes according to a Likert-type scale varying from “1 = extremely low/ineffective” to “7 = extremely high/effective”. Perceived time needed by the transportation sector to completely recover (Part F) according to the scale “1 = less than 6 months”, “2 = between 6 and 12 months”, “3 = between 12 and 18 months”, “4 = between 18 and 24 months”, “5 = more than 24 months”. Mean rating (top number, non-italic) and corresponding standard deviation (bottom number, italic) for each transport mode is presented.
(DOCX)

S2 Table Likelihood ratio chi-square. Likelihood ratio chi-Square for the responses associated to Part D, Part E and Part F according to Negative Binomial Model (NBM).
(DOCX)

S3 Table Deviance/df ratio. Deviance/df ratio for the responses associated to Part D, Part E and Part F according to Negative Binomial Model (NBM).
(DOCX)

## References

[1] WHO. Coronavirus disease (COVID-19) pandemic [Internet]. 2020 [cited 2020 Aug 1]. Available from: https://www.who.int/emergencies/diseases/novel-coronavirus-2019

[2] Huang C, Wang Y, Li X, Ren L, Zhao J, Hu Y, et al Clinical features of patients infected with 2019 novel coronavirus in Wuhan, China. Lancet. 2020;395(10223):497–506. 10.1016/S0140-6736(20)30183-5 31986264 PMC7159299

[3] Sevilla NL. Germs on a plane: the transmission and risks of airplane-borne diseases. Transp Res Rec. 2018;2672(29):93–102. 10.1177/0361198118799709 PMC8282645 34285452

[4] Browne A, Ahmad SS t. O, Beck CR, Nguyen-Van-Tam JS. The roles of transportation and transportation hubs in the propagation of influenza and coronaviruses: a systematic review. J Travel Med. 2016;23(1):1–7. 10.1093/jtm/tav002 26782122 PMC7539332

[5] Wells CR, Sah P, Moghadas SM, Pandey A, Shoukat A, Wang Y, et al Impact of international travel and border control measures on the global spread of the novel 2019 coronavirus outbreak. Proc Natl Acad Sci U S A. 2020;117(13):7504–9. 10.1073/pnas.2002616117 32170017 PMC7132249

[6] Nakamura H, Managi S. Airport risk of importation and exportation of the COVID-19 pandemic. Transp Policy. 2020;96(April):40–7. 10.1016/j.tranpol.2020.06.018 32834679 PMC7328638

[7] WHO. Coronavirus Disease (COVID-19) Dashboard [Internet]. 2020 [cited 2020 Aug 1]. Available from: https://covid19.who.int/

[8] Acter T, Uddin N, Das J, Akhter A, Choudhury TR, Kim S. Evolution of severe acute respiratory syndrome coronavirus 2 (SARS-CoV-2) as coronavirus disease 2019 (COVID-19) pandemic: A global health emergency. Sci Total Environ. 2020;730:138996 10.1016/j.scitotenv.2020.138996 32371230 PMC7190497

[9] Kramer A, Kramer KZ. The potential impact of the Covid-19 pandemic on occupational status, work from home, and occupational mobility. J Vocat Behav. 2020;119(May):1–4. 10.1016/j.jvb.2020.103442 PMC7205621 32390661

[10] Musselwhite C, Avineri E, Susilo Y. Editorial JTH 16 –The Coronavirus disease COVID-19 and implications for transport and health. J Transp Heal. 2020;16(April):4–7. 10.1016/j.jth.2020.100853 32337154 PMC7174824

[11] Troko J, Myles P, Gibson J, Hashim A, Enstone J, Kingdon S, et al Is public transport a risk factor for acute respiratory infection? BMC Infect Dis. 2011;11:2–7. 10.1186/1471-2334-11-2 21235795 PMC3030548

[12] Shen J, Duan H, Zhang B, Wang J, Ji JS, Wang J, et al Prevention and control of COVID-19 in public transportation: Experience from China. Environ Pollut. 2020;266 10.1016/j.envpol.2020.115291 32829124 PMC7833563

[13] Wilder-Smith A, Freedman DO. Isolation, quarantine, social distancing and community containment: Pivotal role for old-style public health measures in the novel coronavirus (2019-nCoV) outbreak. J Travel Med. 2020;27(2):1–4. 10.1093/jtm/taaa020 32052841 PMC7107565

[14] De Vos J. The effect of COVID-19 and subsequent social distancing on travel behavior. Transp Res Interdiscip Perspect. 2020 5;5:100121. 10.1016/j.trip.2020.100121 PMC7180344 34171016

[15] Flaxman S, Mishra S, Gandy A, Unwin HJT, Mellan TA, Coupland H, et al Estimating the effects of non-pharmaceutical interventions on COVID-19 in Europe. Nature. 2020;584(7820):257–61. 10.1038/s41586-020-2405-7 32512579

[16] Lou J, Shen X, Niemeier D. Are stay-at-home orders more difficult to follow for low-income groups? J Transp Geogr. 2020;89(October):102894. 10.1016/j.jtrangeo.2020.102894 PMC7832451 33519126

[17] Shamshiripour A, Rahimi E, Shabanpour R, Mohammadian A (Kouros). How is COVID-19 reshaping activity-travel behavior? Evidence from a comprehensive survey in Chicago. Transp Res Interdiscip Perspect. 2020;7:100216. 10.1016/j.trip.2020.100216 PMC7474875 34173469

[18] Bohte W, Maat K, van Wee B. Measuring attitudes in research on residential self-selection and travel behaviour: A review of theories and empirical research. Transp Rev. 2009;29(3):325–57.

[19] Müggenburg H, Busch-Geertsema A, Lanzendorf M. Mobility biographies: A review of achievements and challenges of the mobility biographies approach and a framework for further research. J Transp Geogr. 2015;46:151–63.

[20] Schoenduwe R, Mueller MG, Peters A, Lanzendorf M. Analysing mobility biographies with the life course calendar: A retrospective survey methodology for longitudinal data collection. J Transp Geogr. 2015;42:98–109.

[21] Ferrer RA, Klein WMP. Risk perceptions and health behavior. Curr Opin Psychol. 2015;5:85–9. 10.1016/j.copsyc.2015.03.012 26258160 PMC4525709

[22] Abdulkareem SA, Augustijn EW, Filatova T, Musial K, Mustafa YT. Risk perception and behavioral change during epidemics: Comparing models of individual and collective learning. PLoS One. 2020;15(1):1–22. 10.1371/journal.pone.0226483 31905206 PMC6944362

[23] Law R. The perceived impact of risks on travel decisions. Int J Tour Res. 2006;8(4):289–300.

[24] Bonaccorsi G, Pierri F, Cinelli M, Porcelli F, Galeazzi A, Flori A, et al Economic and social consequences of human mobility restrictions under COVID-19. Proc Natl Acad Sci. 2020;1–6. 10.1073/pnas.2007658117 32554604 PMC7355033

[25] Mogaji E. Impact of COVID-19 on transportation in Lagos, Nigeria. Transp Res Interdiscip Perspect. 2020;6:100154. 10.1016/j.trip.2020.100154 PMC7290214 34171020

[26] Adekunle IA, Onanuga AT, Akinola OO, Ogunbanjo OW. Modelling spatial variations of coronavirus disease (COVID-19) in Africa. Sci Total Environ. 2020;729:138998 10.1016/j.scitotenv.2020.138998 32361455 PMC7195380

[27] de Haas M, Faber R, Hamersma M. How COVID-19 and the Dutch ‘intelligent lockdown’ change activities, work and travel behaviour: Evidence from longitudinal data in the Netherlands. Transp Res Interdiscip Perspect. 2020;6:100150. 10.1016/j.trip.2020.100150 PMC7284275 34171019

[28] Rogers RW. A protection motivation theory of fear appeals and attitude change. J Psychol. 1975;91(1):93–114. 10.1080/00223980.1975.9915803 28136248

[29] Boer H, Seydel ER. Protection motivation theory. In: Conner M, Norman P, editors. Predicting health behavior second. Maidenhead: Open University Press; 2005 p. 81–126.

[30] Rosenstock IM. The health belief model: Explaining health behavior through expectancies. In: Health Behavior and Health Education. San Francisco: Jossey-Bass; 1990 p. 39–62.

[31] Harrison JA, Mullen PD, Green LW. A meta-analysis of studies of the health belief model with adults. Health Educ Res. 1992;7(1):107–16. 10.1093/her/7.1.107 10148735

[32] Rosenstock IM. Historical origins of the Health Belief Model. Heal Educ Behav. 1974;2(4):328–35.

[33] McKinsey and Company. Global surveys of consumer sentiment during the coronavirus crisis [Internet]. 2020 [cited 2020 Aug 1]. Available from: https://www.mckinsey.com/business-functions/marketing-and-sales/our-insights/global-surveys-of-consumer-sentiment-during-the-coronavirus-crisis

[34] Belot M, Papageorge NW, Choi S, Tripodi E, Jamison JC, van den Broek-Altenburg E. Six-country survey on COVID-19. Bonn; 2020.

[35] Jones SP. COVID-19 behaviour tracker [Internet]. 2020 [cited 2020 Aug 1]. Available from: https://public.tableau.com/profile/ighi#!/vizhome/ICLYouGovCovid-19Tracker_V0_3/1Specificpreventativebehaviourbycountry

[36] Fetzer T, Witte M, Hensel L, Jachimowicz JM, Haushofer J, Ivchenko A, et al. Perceptions of an insufficient government response at the onset of the COVID-19 pandemic are associated with lower mental well-being. Boston; 2020.

[37] Stockemer D. Quantitative Methods for the Social Sciences. Vol. 50 Cham: Springer; 2019 185 p.

[38] Barbieri DM, Lou B, Passavanti M, Hui C, Antunes D, Maharaj B, et al A survey dataset to evaluate the changes in mobility and transportation due to COVID-19 travel restrictions in Australia, Brazil, China, Ghana, India, Iran, Italy, Norway, South Africa, United States. Data Br. 2020;33:106459 10.1016/j.dib.2020.106459 33163599 PMC7607379

[39] Brislin RW. Comparative research methodology: Cross-cultural studies. Int J Psychol. 1976;11(3).

[40] Brög W. Surveys on daily mobility are not “surveys to go.” Transp Res Procedia. 2015;11:98–107.

[41] De Beuckelaer A, Lievens F. Measurement equivalence of paper-and-pencil and internet organisational surveys: A large scale examination in 16 countries. Appl Psychol. 2009;58(2):336–61.

[42] Oxford University. Coronavirus government response tracker [Internet]. 2020 [cited 2020 Aug 1]. Available from: https://www.bsg.ox.ac.uk/research/research-projects/coronavirus-government-response-tracker

[43] Coughlin SS. Recall bias in epidemiologic studies. J Clin Epidemiol. 1990;43(1):87–91. 10.1016/0895-4356(90)90060-3 2319285

[44] Jaspers E, Lubbers M, De Graaf ND. Measuring once twice: An evaluation of recalling attitudes in survey research. Eur Sociol Rev. 2009;25(3):287–301.

[45] Solga H. Longitudinal surveys and the study of occupational mobility: Panel and retrospective design in comparison. Qual Quant. 2001;35(3):291–309.

[46] Barsky AJ. Forgetting, fabricating, and telescoping. Arch Intern Med. 2002;162(9):981 10.1001/archinte.162.9.981 11996606

[47] Hipp L, Bünning M, Munnes S, Sauermann A. Problems and pitfalls of retrospective survey questions in COVID-19 studies. Surv Res Methods. 2020;14(2):109–14.

[48] Smith TW. Recalling attitudes: An analysis of retrospective questions on the 1982 GSS. Public Opin Q. 1984;48(3):639–49.

[49] Cartenì A, Di Francesco L, Martino M. How mobility habits influenced the spread of the COVID-19 pandemic: Results from the Italian case study. Sci Total Environ. 2020;741 10.1016/j.scitotenv.2020.140489 32599395 PMC7313484

[50] Bish A, Michie S. Demographic and attitudinal determinants of protective behaviours during a pandemic: A review. Br J Health Psychol. 2010;15(4):797–824. 10.1348/135910710X485826 20109274 PMC7185452

[51] World Population Review. Gini coefficient by country 2020 [Internet]. 2020 [cited 2020 Aug 1]. Available from: https://worldpopulationreview.com/country-rankings/gini-coefficient-by-country

[52] World Economic Forum. The Inclusive Development Index 2018 summary and data highlights. Geneva; 2018.

[53] United Nations Development Programme. Human Development Report 2019. New York; 2019.

[54] Ajide KB, Ibrahim RL, Alimi OY. Estimating the impacts of lockdown on Covid-19 cases in Nigeria. Transp Res Interdiscip Perspect. 2020;7(June):100217. 10.1016/j.trip.2020.100217 PMC7474887 34173470

[55] Oztig LI, Askin OE. Human mobility and coronavirus disease 2019 (COVID-19): a negative binomial regression analysis. Public Health. 2020;185:364–7. 10.1016/j.puhe.2020.07.002 32739776 PMC7351378

[56] Sax LJ, Gilmartin SK, Bryant AN. Assessing response rates and nonresponse bias in web and paper surveys. Res High Educ. 2003;44(4):409–32.

[57] Kashfi SA, Bunker JM, Yigitcanlar T. Understanding the effects of complex seasonality on suburban daily transit ridership. J Transp Geogr. 2015;46:67–80.

[58] Tucker P, Gilliland J. The effect of season and weather on physical activity: A systematic review. Public Health. 2007;121(12):909–22. 10.1016/j.puhe.2007.04.009 17920646

[59] Ghana government via Wikimedia Commons. New Ghana Map 2019 [Internet]. 2019 [cited 2020 Dec 15]. Available from: https://en.wikipedia.org/wiki/File:New_Ghana_Map_2019.jpg

[60] Newfraferz87 via Wikimedia Commons. China blank province map [Internet]. 2019 [cited 2020 Dec 15]. Available from: https://en.wikipedia.org/wiki/File:China_blank_province_map.svg

[61] Prateek01~commonswiki via Wikimedia Commons. India ter1 [Internet]. 2006 [cited 2020 Dec 15]. Available from: https://commons.wikimedia.org/wiki/File:India_ter1.jpg

[62] Amada44 via Wikimedia Commons. Map of South Africa [Internet]. 2009 [cited 2020 Dec 15]. Available from: https://en.wikipedia.org/wiki/File:Map_of_South_Africa.svg

[63] Nick Carson via Wikimedia Commons. Map of the 2009 Southern Australia heat wave affected area [Internet]. 2014 [cited 2020 Dec 15]. Available from: http://www.publicdomainfiles.com/show_file.php?id=13935648615025

[64] Rarelibra via Wikimedia Commons. Brazil Municipalities [Internet]. 2006 [cited 2020 Dec 15]. Available from: https://commons.wikimedia.org/wiki/File:Brazil_Municipalities.png

[65] Siamax via Wikimedia Commons. Blank-Map-Iran [Internet]. 2007 [cited 2020 Dec 15]. Available from: https://commons.wikimedia.org/wiki/File:Blank-Map-Iran.PNG

[66] Furfur via Wikimedia Commons. Nye fylker—regjeringen [Internet]. 2020 [cited 2020 Dec 15]. Available from: https://commons.wikimedia.org/wiki/File:Nye_fylker_-_regjeringen.no.svg

[67] Kaboom88 via Wikimedia Commons. Blank US map borders [Internet]. 2007 [cited 2020 Dec 15]. Available from: https://commons.wikimedia.org/wiki/File:Blank_US_map_borders.svg

[68] Flanker via Wikimedia Commons. Map of Italy blank [Internet]. 2006 [cited 2020 Dec 15]. Available from: https://commons.wikimedia.org/wiki/File:Map_of_Italy_blank.svg

[69] Arimura M, Ha TV, Okumura K, Asada T. Changes in urban mobility in Sapporo city, Japan due to the Covid-19 emergency declarations. Transp Res Interdiscip Perspect. 2020;7:100212. 10.1016/j.trip.2020.100212 PMC7834002 34173468

[70] Beck MJ, Hensher DA. Insights into the impact of COVID-19 on household travel and activities in Australia–The early days under restrictions. Transp Policy. 2020;96(May):76–93. 10.1016/j.tranpol.2020.07.001 32834680 PMC7330570

[71] Pucher J, Peng ZR, Mittal N, Zhu Y, Korattyswaroopam N. Urban transport trends and policies in China and India: Impacts of rapid economic growth. Transp Rev. 2007;27(4):379–410.

[72] Pearce B. The shape of air travel markets over the next 20 years. In: Global Airport Development Conference. Athens; 2014.

[73] Cahyanto I, Wiblishauser M, Pennington-Gray L, Schroeder A. The dynamics of travel avoidance: The case of Ebola in the U.S. Tour Manag Perspect. 2016;20:195–203. 10.1016/j.tmp.2016.09.004 32289007 PMC7147605

[74] Wilder-Smith A. The severe acute respiratory syndrome: Impact on travel and tourism. Travel Med Infect Dis. 2006;4(2):53–60. 10.1016/j.tmaid.2005.04.004 16887725 PMC7106206

[75] Gössling S, Scott D, Hall CM. Pandemics, tourism and global change: a rapid assessment of COVID-19. J Sustain Tour. 2020;1–20.

[76] Parady G, Taniguchi A, Takami K. Travel behavior changes during the COVID-19 pandemic in Japan: Analyzing the effects of risk perception and social in fl uence on going-out self-restriction. Transp Res Interdiscip Perspect. 2020;7:100181. 10.1016/j.trip.2022.100649 PMC9239539 35782787

[77] Borkowski P, Jażdżewska-Gutta M, Szmelter-Jarosz A. Lockdowned: Everyday mobility changes in response to COVID-19. J Transp Geogr. 2021;90(November 2020). 10.1016/j.jtrangeo.2020.102906 PMC9188832 35721765

[78] Google. COVID-19 Community Mobility Reports [Internet]. 2020 [cited 2020 Aug 1]. Available from: https://www.google.com/covid19/mobility/

[79] Apple. Mobility Trends Reports [Internet]. 2020 [cited 2020 Aug 1]. Available from: https://www.apple.com/covid19/mobility

[80] Huang X, Li Z, Jiang Y, Li X, Porter D. Twitter reveals human mobility dynamics during the COVID-19 pandemic. PLoS One. 2020;15(11 November):1–21. 10.1371/journal.pone.0241957 33170889 PMC7654838

[81] Sadique MZ, Edmunds WJ, Smith RD, Meerding WJ, De Zwart O, Brug J, et al Precautionary behavior in response to perceived threat of pandemic influenza. Emerg Infect Dis. 2007;13(9):1307–13. 10.3201/eid1309.070372 18252100 PMC2857294

[82] Antonovsky A. Social class, life expectancy and overall mortality. Milbank Q. 1967;45(2):31–73. 6034566

[83] Mcisaac SJ, Wilkinson RG. Income distribution and cause-specific mortality. Eur J Public Health. 1997;7(1):45–53.

[84] Wilkinson RG. Income distribution and life expectancy. Br Med J. 1992;304(6828):165–8. 10.1136/bmj.304.6820.165 1637372 PMC1881178

[85] Feinstein JS. The relationship between socioeconomic status and health a review of the literature. Milbank Q. 1993;71(2):279–322. 8510603

[86] Adler NE, Boyce T, Chesney MA, Cohen S, Folkman S, Kahn RL, et al Socioeconomic status and health: the challenge of the gradient. Am Psychol. 1994;49(1):15–24. 10.1037//0003-066x.49.1.15 8122813

[87] Soobader MJ, LeClere FB. Aggregation and the measurement of income inequality: effects on morbidity. Soc Sci Med. 1999;48(6):733–44. 10.1016/s0277-9536(98)00401-8 10190636

[88] Lynch JW, Smith GD, Kaplan GA, House JS. Income inequality and mortality: Importance to health of individual income, psychosocial environment, or material conditions. Br Med J. 2000;320(7243):1200–4. 10.1136/bmj.320.7243.1200 10784551 PMC1127589

[89] Wilkinson RG, Pickett KE. Income inequality and population health: A review and explanation of the evidence. Soc Sci Med. 2006;62(7):1768–84. 10.1016/j.socscimed.2005.08.036 16226363

[90] Slovic P. Risk, society, and policy series.The perception of risk. London: Earthscan Publications; 2000.

[91] Piot P, Greener R, Russell S. Squaring the circle: AIDS, poverty, and human development. PLoS Med. 2007;4(10):1571–5. 10.1371/journal.pmed.0040314 17958469 PMC2039763

[92] Peretti-Watel P, Seror V, Verger P, Guignard R, Legleye S, Beck F. Smokers’ risk perception, socioeconomic status and source of information on cancer. Addict Behav. 2014;39(9):1304–10. 10.1016/j.addbeh.2014.04.016 24836161

[93] Ibuka Y, Chapman GB, Meyers LA, Li M, Galvani AP. The dynamics of risk perceptions and precautionary behavior in response to 2009 (H1N1) pandemic influenza. BMC Infect Dis. 2010;10(1):296 10.1186/1471-2334-10-296 20946662 PMC2964717

[94] Schmiege SJ, Bryan A, Klein WMP. Distinctions between worry and perceived risk in the context of the theory of planned behavior. J Appl Soc Psychol. 2009;39(1):95–119.

[95] Quintal VA, Lee JA, Soutar GN. Risk, uncertainty and the theory of planned behavior: A tourism example. Tour Manag. 2010;31(6):797–805.

[96] Weill JA, Stigler M, Deschenes O, Springborn MR. Social distancing responses to COVID-19 emergency declarations strongly differentiated by income. Proc Natl Acad Sci U S A. 2020;117(33):19658–60. 10.1073/pnas.2009412117 32727905 PMC7443940

[97] Verlinghieri E, Schwanen T. Transport and mobility justice: Evolving discussions. J Transp Geogr. 2020;87(June). 10.1016/j.jtrangeo.2020.102798 32834675 PMC7359804

[98] Nordgren LF, van Der Pligt J, van Harreveld F. Unpacking perceived control in risk perception: The mediating role of anticipated regret. J Behav Decis Mak. 2007;20:533–44.

[99] Gustafson PE. Gender differences in risk perception: Theoretical and methodological perspectives. Risk Anal. 1998;18(6):805–11. 10.1023/b:rian.0000005926.03250.c0 9972583

[100] Hotle S, Murray-Tuite P, Singh K. Influenza risk perception and travel-related health protection behavior in the US: Insights for the aftermath of the COVID-19 outbreak. Transp Res Interdiscip Perspect. 2020;5:100127. 10.1016/j.trip.2020.100127 PMC7211591 34171017

[101] Budd L, Ison S. Responsible Transport: A post-COVID agenda for transport policy and practice. Transp Res Interdiscip Perspect. 2020;6:100151. 10.1016/j.trip.2020.100151 PMC7311912 34173454

[102] Kuzemko C, Bradshaw M, Bridge G, Goldthau A, Jewell J, Overland I, et al Covid-19 and the politics of sustainable energy transitions. Energy Res Soc Sci. 2020;68(July):101685 10.1016/j.erss.2020.101685 32839704 PMC7330551

