# Supplementary material for: Impact of COVID-19 pandemic on mobility in ten countries and associated perceived risk for all transport modes
Source: PLoS One. 2021 Feb 1;16(2):e0245886. doi: 10.1371/journal.pone.0245886 (PMC7850470; doi:10.1371/journal.pone.0245886)
Supplement: S1 Table — Perceived probability of contracting COVID-19 (Part D) and perceived effectiveness of curbing COVID-19 (Part E) for transport modes according to a Likert-type scale varying from “1 = extremely low/ineffective” to “7 = extremely high/effective”. Perceived time needed by the transportation sector to completely recover (Part F) according to the scale “1 = less than 6 months”, “2 = between 6 and 12 months”, “3 = between 12 and 18 months”, “4 = between 18 and 24 months”, “5 = more than 24 months”. Mean rating (top number, non-italic) and corresponding standard deviation (bottom number, italic) for each transport mode is presented. (DOCX) [file pone.0245886.s001.docx]

**S1 Table.** **Perceptions encompassing mobility and pandemic.**

| ***Part***  ***D*** | **Walk** | **Bi**  **-cycle** | **Motor**  **-cycle** | **Car alone** | **Car shared** | **Bus** | **Metro/**  **Tram** | **Train** | **Air**  **-plane** |
| --- | --- | --- | --- | --- | --- | --- | --- | --- | --- |
| **IT** | 2.58 | 2.04 | 1.79 | 1.45 | 3.19 | 5.36 | 5.39 | 5.27 | 5.32 |
|  | *1.39* | *1.16* | *1.07* | *0.85* | *1.39* | *1.32* | *1.26* | *1.31* | *1.31* |
| **USA** | 2.60 | 2.20 | 2.13 | 1.77 | 3.45 | 5.15 | 5.34 | 5.27 | 5.61 |
|  | *1.47* | *1.34* | *1.34* | *1.35* | *1.54* | *1.53* | *1.51* | *1.50* | *1.49* |
| **IR** | 3.60 | 3.28 | 3.31 | 2.56 | 6.00 | 6.46 | 6.22 | 6.30 | 6.14 |
|  | *1.63* | *1.43* | *1.45* | *1.43* | *1.32* | *1.18* | *1.36* | *1.24* | *1.25* |
| **NO** | 2.47 | 1.85 | 1.58 | 1.32 | 3.27 | 4.54 | 4.67 | 4.45 | 5.01 |
|  | *1.22* | *0.98* | *0.88* | *0.69* | *1.34* | *1.33* | *1.27* | *1.31* | *1.35* |
| **BR** | 3.59 | 3.02 | 2.83 | 2.20 | 3.89 | 6.13 | 6.16 | 6.06 | 5.99 |
|  | *1.49* | *1.37* | *1.34* | *1.14* | *1.38* | *1.26* | *1.20* | *1.23* | *1.24* |
| **ZA** | 3.63 | 2.60 | 2.49 | 1.80 | 3.58 | 5.80 | 5.19 | 5.84 | 5.80 |
|  | *1.81* | *1.36* | *1.38* | *1.04* | *1.41* | *1.42* | *1.56* | *1.48* | *1.57* |
| **AU** | 3.01 | 2.14 | 1.97 | 1.56 | 3.17 | 4.90 | 4.97 | 5.07 | 5.55 |
|  | *1.57* | *1.27* | *1.23* | *1.10* | *1.42* | *1.59* | *1.55* | *1.52* | *1.55* |
| **IN** | 3.34 | 2.85 | 3.05 | 2.49 | 3.80 | 5.19 | 4.81 | 5.31 | 5.08 |
|  | *1.81* | *1.50* | *1.52* | *1.51* | *1.60* | *2.14* | *2.00* | *2.00* | *1.93* |
| **CH** | 3.47 | 3.24 | 3.26 | 2.95 | 4.54 | 5.27 | 5.03 | 5.30 | 5.19 |
|  | *1.78* | *1.69* | *1.62* | *1.66* | *1.42* | *1.55* | *1.70* | *1.66* | *1.67* |
| **GH** | 3.61 | 2.69 | 3.19 | 2.03 | 3.91 | 5.23 | 3.78 | 4.24 | 4.50 |
|  | *1.83* | *1.50* | *1.65* | *1.34* | *1.61* | *1.89* | *1.76* | *1.86* | *1.88* |

*(continue)*

| ***Part***  ***E*** | **Walk** | **Bi**  **-cycle** | **Motor**  **-cycle** | **Car alone** | **Car shared** | **Bus** | **Metro/**  **Tram** | **Train** | **Air**  **-plane** |
| --- | --- | --- | --- | --- | --- | --- | --- | --- | --- |
| **IT** | 4.42 | 4.43 | 4.49 | 4.59 | 4.36 | 4.03 | 3.95 | 4.10 | 4.21 |
|  | *1.70* | *1.70* | *1.66* | *1.70* | *1.47* | *1.74* | *1.69* | *1.60* | *1.65* |
| **USA** | 4.21 | 4.18 | 4.17 | 4.31 | 4.28 | 4.33 | 4.31 | 4.37 | 4.29 |
|  | *1.50* | *1.49* | *1.46* | *1.53* | *1.45* | *1.46* | *1.53* | *1.46* | *1.54* |
| **IR** | 4.66 | 4.43 | 4.40 | 4.28 | 5.97 | 6.15 | 5.69 | 6.14 | 6.09 |
|  | *1.76* | *1.69* | *1.67* | *1.90* | *1.54* | *1.58* | *1.74* | *1.48* | *1.43* |
| **NO** | 4.16 | 4.10 | 4.01 | 4.10 | 4.17 | 4.55 | 4.63 | 4.62 | 4.71 |
|  | *1.75* | *1.71* | *1.72* | *1.84* | *1.21* | *1.30* | *1.23* | *1.15* | *1.41* |
| **BR** | 3.59 | 3.51 | 3.55 | 3.63 | 3.74 | 3.74 | 3.72 | 3.82 | 4.24 |
|  | *1.58* | *1.53* | *1.51* | *1.56* | *1.57* | *1.92* | *1.81* | *1.81* | *1.68* |
| **ZA** | 3.74 | 3.92 | 4.03 | 4.30 | 4.19 | 3.98 | 4.29 | 4.49 | 5.21 |
|  | *1.70* | *1.62* | *1.59* | *1.71* | *1.53* | *1.81* | *1.72* | *1.80* | *1.86* |
| **AU** | 4.78 | 4.77 | 4.74 | 4.86 | 4.75 | 4.54 | 4.52 | 4.49 | 5.11 |
|  | *1.39* | *1.39* | *1.43* | *1.51* | *1.40* | *1.47* | *1.40* | *1.41* | *1.59* |
| **IN** | 4.08 | 4.17 | 4.39 | 4.57 | 4.66 | 4.88 | 4.67 | 4.98 | 5.05 |
|  | *1.75* | *1.66* | *1.62* | *1.67* | *1.73* | *2.14* | *2.04* | *2.09* | *2.05* |
| **CH** | 4.41 | 4.34 | 4.39 | 4.33 | 4.95 | 5.12 | 5.28 | 5.30 | 5.26 |
|  | *1.78* | *1.75* | *1.72* | *1.85* | *1.61* | *1.87* | *1.60* | *1.75* | *1.81* |
| **GH** | 3.52 | 3.52 | 3.73 | 3.99 | 4.01 | 3.94 | 3.15 | 3.32 | 3.99 |
|  | *1.76* | *1.70* | *1.74* | *1.89* | *1.77* | *1.99* | *1.67* | *1.76* | *1.98* |
| ***Part***  ***F*** | **In the**  **region** | **In the**  **country** | **In the**  **world** |  |  |  |  |  |  |
| **IT** | 2.62 | 3.09 | 3.83 |  |  |  |  |  |  |
|  | *1.25* | *1.21* | *1.31* |  |  |  |  |  |  |
| **USA** | 2.88 | 3.35 | 3.85 |  |  |  |  |  |  |
|  | *1.46* | *1.41* | *1.43* |  |  |  |  |  |  |
| **IR** | 2.93 | 3.42 | 3.76 |  |  |  |  |  |  |
|  | *1.20* | *1.31* | *1.28* |  |  |  |  |  |  |
| **NO** | 2.46 | 2.92 | 4.16 |  |  |  |  |  |  |
|  | *1.24* | *1.23* | *1.25* |  |  |  |  |  |  |
| **BR** | 2.36 | 2.88 | 3.22 |  |  |  |  |  |  |
|  | *1.23* | *1.28* | *1.42* |  |  |  |  |  |  |
| **ZA** | 2.40 | 2.91 | 3.65 |  |  |  |  |  |  |
|  | *1.22* | *1.27* | *1.45* |  |  |  |  |  |  |
| **AU** | 2.16 | 2.71 | 4.09 |  |  |  |  |  |  |
|  | *1.26* | *1.24* | *1.34* |  |  |  |  |  |  |
| **IN** | 2.08 | 2.55 | 3.10 |  |  |  |  |  |  |
|  | *1.20* | *1.24* | *1.39* |  |  |  |  |  |  |
| **CH** | 2.04 | 2.34 | 3.55 |  |  |  |  |  |  |
|  | *1.48* | *1.48* | *1.54* |  |  |  |  |  |  |
| **GH** | 2.28 | 2.58 | 3.29 |  |  |  |  |  |  |
|  | *1.34* | *1.33* | *1.45* |  |  |  |  |  |  |

Perceived probability of contracting COVID-19 (Part D) and perceived effectiveness of curbing COVID-19 (Part E) for transport modes according to a Likert-type scale varying from “1=extremely low/ineffective” to “7=extremely high/effective”. Perceived time needed by the transportation sector to completely recover (Part F) according to the scale “1=less than 6 months”, “2= between 6 and 12 months”, “3=between 12 and 18 months”, “4=between 18 and 24 months”, “5=more than 24 months”. Mean rating (top number, non-italic) and corresponding standard deviation (bottom number, italic) for each transport mode is presented.
