# Supplementary material for: Impact of COVID-19 pandemic on mobility in ten countries and associated perceived risk for all transport modes
Source: PLoS One. 2021 Feb 1;16(2):e0245886. doi: 10.1371/journal.pone.0245886 (PMC7850470; doi:10.1371/journal.pone.0245886)
Supplement: S2 Table — Likelihood ratio chi-Square for the responses associated to Part D, Part E and Part F according to Negative Binomial Model (NBM). (DOCX) [file pone.0245886.s002.docx]

**S2 Table.** **Likelihood ratio chi-Square.**

| **Part D** | | | | | | | | | | | | | | | | | | | | | | | | | | | | | |
| --- | --- | --- | --- | --- | --- | --- | --- | --- | --- | --- | --- | --- | --- | --- | --- | --- | --- | --- | --- | --- | --- | --- | --- | --- | --- | --- | --- | --- | --- |
| **Walk** | **Bicycle** | | **Motor**  **-cycle** | | | **Car alone** | | | | **Car shared** | | | | **Bus** | | | | **Metro/**  **Tram** | | | | **Train** | | | | **Airplane** | | | |
| 179.03 | 216.12 | | 343.49 | | | 314.42 | | | | 150.06 | | | | 45.24 | | | | 29.63 | | | | 29.84 | | | | 23.47 | | | |
| **Part E** | | | | | | | | | | | | | | | | | | | | | | | | | | | | | |
| **Walk** | **Bicycle** | | | **Motor**  **-cycle** | | | **Car alone** | | | | **Car shared** | | | | **Bus** | | | | **Metro/**  **Tram** | | | | **Train** | | | | **Airplane** | | |
| 12.56 | 11.68 | | | 12.99 | | | 13.73 | | | | 23.03 | | | | 37.37 | | | | 18.03 | | | | 29.52 | | | | 39.38 | | |
| **Part F** | | | | | | | |  | | | |  | | | |  | | | |  | | | |  | | | |  | |
| **In the**  **region** | | **In the country** | | | **In the world** | | | |  | | | |  | | | |  | | | |  | | | |  | | | |  |
| 98.50 | | 114.33 | | | 67.39 | | | |  | | | |  | | | |  | | | |  | | | |  | | | |  |

Likelihood ratio chi-Square for the responses associated to Part D, Part E and Part F according to Negative Binomial Model (NBM).
