# Supplementary material for: Impact of COVID-19 pandemic on mobility in ten countries and associated perceived risk for all transport modes
Source: PLoS One. 2021 Feb 1;16(2):e0245886. doi: 10.1371/journal.pone.0245886 (PMC7850470; doi:10.1371/journal.pone.0245886)
Supplement: S3 Table — Deviance/df ratio for the responses associated to Part D, Part E and Part F according to Negative Binomial Model (NBM). (DOCX) [file pone.0245886.s003.docx]

**S3 Table.** **Deviance/df ratio.**

| **Part D** | | | | | | | | | | | | | | | | | | | | | | |  |
| --- | --- | --- | --- | --- | --- | --- | --- | --- | --- | --- | --- | --- | --- | --- | --- | --- | --- | --- | --- | --- | --- | --- | --- |
| **Walk** | **Bicycle** | | **Motor**  **-cycle** | | **Car alone** | | | **Car shared** | | | **Bus** | | | **Metro/**  **Tram** | | | **Train** | | | **Airplane** | | | |
| 0.216 | 0.209 | | 0.205 | | 0.225 | | | 0.152 | | | 0.115 | | | 0.123 | | | 0.114 | | | 0.109 | | | |
| **Part E** | | | | | | | | | | | | | | | | | | | | | | |  |
| **Walk** | **Bicycle** | | **Motor**  **-cycle** | | **Car alone** | | | **Car shared** | | | **Bus** | | | **Metro/**  **Tram** | | | **Train** | | | **Airplane** | | | |
| 0.187 | 0.175 | | 0.166 | | 0.183 | | | 0.145 | | | 0.188 | | | 0.172 | | | 0.166 | | | 0.161 | | | |
| **Part F** | | | | | |  | | |  | | |  | | |  | | |  | | |  | |  |
| **In the**  **region** | | **In the country** | | **In the world** | | |  | | |  | | |  | | |  | | |  | | |  |  |
| 0.183 | | 0.193 | | 0.185 | | |  | | |  | | |  | | |  | | |  | | |  |  |

Deviance/df ratio for the responses associated to Part D, Part E and Part F according to Negative Binomial Model (NBM).
